# Hospital-associated methicillin-resistant *Staphylococcus aureus *(HA-MRSA) in Italy

**DOI:** 10.1186/1476-0711-8-22

**Published:** 2009-06-24

**Authors:** Floriana Campanile, Dafne Bongiorno, Sonia Borbone, Stefania Stefani

**Affiliations:** 1 Department of Microbiology, University of Catania, Italy

## Abstract

The aim of our study was to trace the dynamic changes of hospital-associated methicillin-resistant *Staphylococcus aureus *(HA-MRSA) lineages in Italy, comparing the genotypic backgrounds of contemporary isolates over a period of 17 years, with those of a sample of early MRSA strains from 1980.

In total, 301 non-repetitive MRSA clinical isolates, recovered from 19 Italian hospitals between 1990 and 2007 were selected and analyzed for their antibiotic resistance, typed by PFGE and SCC*mec*, grouped into clonal-types and further characterized using Multi Locus Sequence Typing (MLST). A sample of fifteen early MRSA strains from 1980 was also used for comparison.

The most interesting feature was the recent increase of ST228-MRSA-I (formerly the Italian clone; PFGE E) over the period 2000–2007 (57%), when compared to the period 1990–1999 (29%), and its stability to date, associated with a decrease of the highly epidemic ST247-MRSA-IA (formerly the Iberian clone; PFGE A), (23% from 1990 to 1999, 6% from 2000 to 2007). ST1-MRSA-I (1 out of 2 strains carrying *ccr*A_2_B_2_), ST8-MRSA-I (4 strains), ST15-MRSA-I (1 out of 4 carrying *ccr*A_2_B_2_) and ST30-MRSA-I (2 out of 5 carrying no *ccr*AB-types and *ccr*C) were the predominant earliest STs among the MRSA strains in 1980.

A temporal shift in the susceptibility levels to glycopeptides was observed: strains with vancomycin MIC of ≥ 2 mg/L increased from 19.4% to 35.5%.

In conclusion, we describe the alternation of MRSA clones that occurred in hospitals from 1990 to 2007 and the increase of the glycopeptide MIC levels, reflecting a worldwide trend. We document the detection of ST1, ST8, ST15 and ST30 in the 1980 isolates; we hypothesize their possible latency and their appearance as the current CA-MRSA clones.

## Introduction

Among EU countries, Italy, together with Spain, Greece, Portugal, and Great Britain, has a high frequency of isolation of methicillin-resistant *Staphylococcus aureus *(MRSA) in hospitals. After an initial and continuous increase between 1994 and 2001 [1], the annual reports from EARSS (1999–2007) described the isolation of MRSA from bloodstream infections in 1999 as 40%, but, unlike the trend in other European countries such as Great Britain or Greece, a constant small average decrement was registered in the 2001–2007 period, reaching an average value of 33.7% in 2007 .

These data are only partially representative of the Italian nosocomial MRSA prevalence. MRSA is responsible for various infections and considerable variations between institutions and wards, often in the same geographical areas, exist, demonstrating that MRSA prevalence, in some settings, significantly exceeds previous estimates [1], sometimes accounting for approximately 40–60% of all hospital acquired strains [2-5]. There could be many explanations for these differences: infection control measures, antibiotic prophylaxis and treatments used in each ward/hospital and, not less important, the clonal and often epidemic nature of these microrganisms. Certain strains can be found worldwide yet only few clones are responsible for most MRSA infections [1,6,7].

Analysis of more than 3,000 isolates from Southern Europe, the United States, and South America, showed that nearly 70% of them belonged to five major pandemic clones, namely the Iberian (ST 247-MRSA-IA), Brazilian (ST239-MRSA-IIIA), Hungarian (ST239-MRSA-III), New York/Japan (ST5-MRSA-II), and Pediatric (ST5-MRSA-IV) clones [8]. Dominant and minor lineages constitute the *S.aureus *population [9]. Each lineage is remarkably distinct and isolates of the same lineage, but from diverse geographical locations or from different periods, can be remarkably similar (except for their mobile genetic element – MGE – contents that account for 10–20% of the *S.aureus *chromosome) [10,11]. Evolution of lineages happened independently by different mechanisms [12] and MGEs encoding virulence and resistance genes can move into and out of strains.

In recent years, the widespread use of antibiotics has undoubtedly accelerated the evolution of *S.aureus*, and led to the emergence of strains that have systematically acquired multiple resistance genes [1,13]. With the current emergence of multi-drug resistant isolates in hospitals on the one hand [14] and the dramatically increased incidence of hyper-virulent community-associated MRSA (CA-MRSA) on the other [15,16], MRSA has been able to evolve rapidly and create new clinical problems.

A greater understanding of how the bacteria have evolved in any geographical area can help us to rapidly identify new outbreak strains and even prevent emergence and diffusion of more resistant clones [17].

There have been only few studies reporting the dynamics of MRSA over long periods of time [18-23]. In almost all these studies, despite the worldwide predominance of a few MRSA clones, the authors demonstrated local strain diversity and reported that predominant MRSA strains seemed to change over time and emerging clones were appearing or replacing the oldest.

The aim of our study was to trace the dynamics of genotypic changes and the shifts in the levels of susceptibility to antibiotics, including glycopeptides, of MRSA lineages in Italy, comparing the genotypic backgrounds of contemporary isolates with those of a sample of early MRSA strains from 1980. The study also provides a complete overview of predominant and sporadic MRSA strains in our country, over a period of 17 years, from two surveillances projects (1990–1998 and 2000 – 2007).

## Methods

### Isolates

Three hundred and one MRSA clinical isolates, recovered from 19 Italian hospitals between 1990 and 2007 were selected from two *Staphylococcus aureus *collections, which were sent to our laboratory during previous multi-centre studies. The first collection (called A throughout the text) of 160 strains was selected as non-repetitive isolates representative of the different parts of Italy from among 426 methicillin-resistant *S.aureus *(MRSA) strains isolated in the period 1990–1999 [24]. The second group of 141 strains (called B), were isolated in the period 2000–2007, and were selected with the same criteria described above, from among approximately 1,000 different *S.aureus *isolates [25].

Both groups of isolates were from various clinical specimens and in particular, group A: 37% of cases from lower respiratory tract specimens, 29% from blood cultures (7% associated to a CVC), 1% from cerebrospinal fluid, and 31% from SSSIs. Group B isolates were almost comparable, with a greater percentage only for lower respiratory tract specimens (53%), 19% from blood cultures, 3% from cerebrospinal fluid and 25% from SSSIs.

A sample of fifteen early MRSA strains from 1980 was used for comparison [26].

All isolates were re-identified at the species level by catalase test, *S. aureus *agglutination test (STAPHYLASE TEST; Oxoid Ltd. Basingstoke, Hampshire, UK) and biochemical tests (API-Staph system; bio Mérieux SA, Marcy-l'Etoile, France). All strains were stored at -80°C until use. Methicillin-resistance was evaluated by disk diffusion method, and correlated to the presence of the *mec*A gene (see below).

The distribution of strains throughout Italy was, in both projects, approximately 20% from hospitals in Northern Italy, 35% from the center, and 45% from Southern Italy.

### *In vitro *susceptibility testing of antibiotics

The antimicrobial susceptibility profiles of the 301 MRSA strains to the main classes of antibiotics were determined by using the Kirby-Bauer method, according to CLSI guidelines [27]. The MRSA isolates were tested against a panel of 9 antimicrobial agents as follows: oxacillin-1 μg, ciprofloxacin-5 μg, chloramphenicol-30 μg, gentamicin-10 μg, erythromycin-15 μg, clindamycin-2 μg, trimethoprim-sulfamethoxazole-25 μg, rifampin-5 μg and tetracycline-30 μg.

*In vitro *susceptibility testing for vancomycin, teicoplanin, quinupristin/dalfopristin, linezolid, tigecycline and daptomycin was further performed by the broth microdilution method to determine the minimum inhibitory concentrations (MICs), following the CLSI guidelines [27]. All antibiotics were kindly provided, as standard reference powders, by the following manufacturers: daptomycin from Novartis (Basel, CH); linezolid from Pfizer (Groton, CT, USA); quinupristin/dalfopristin and teicoplanin from Aventis (West Malling, UK). Vancomycin was from Sigma Chemical (St. Louis, MO, USA).

MICs were determined using Muller-Hinton broth (Oxoid, Milan, Italy), and for daptomycin tests the broth was supplemented to yield a final concentration of 50 mg/L calcium. *S. aureus *ATCC 29213 was used as quality control. Results were read after incubation at 37°C for 18–24 h. Susceptibility to daptomycin was defined as a MIC value of ≤ 1 mg/L; CLSI guideline MIC breakpoints were used for all the other antibiotics tested [27].

### Pulsed-field gel electrophoresis (PFGE)

Genomic DNA was prepared in agarose plugs as previously described [28]. DNA was digested with 20 U of *Sma*I (BioLabs, New England, USA) at 30°C overnight. PFGE was carried out in a CHEF-DR II apparatus; (Bio-Rad, CA, USA). Macrorestriction fragments were separated on 1% (wt/vol) ultra pure agarose gels (Sigma Aldrich, S.Louis, MO, USA) at 6 V/cm for 21 h at 14°C, with pulse times of 5 s – 35 s, to separate *Sma*I patterns. Lambda DNA concatemers (New England BioLabs, Beverly, MA, USA) were used as molecular size markers.

Similarities among macrorestriction patterns were previously identified according to established criteria [29] in each of the three time frames. The comparison of similarities among groups for a long-term evaluation was performed by using the type strain of each PFGE profile of each time frame as internal standard.

### PCR and SCCmec typing

Genomic DNA was extracted from all bacterial cultures and used as a template for amplification. All isolates were screened for the presence of the *mec*A gene. PCR experiments were carried out following procedures previously published [28]. PCR products were analyzed by electrophoresis on 1% agarose gels (Sigma Aldrich, Saint Louis Mo, USA).

The amplification procedure for the detection of the Panton-Valentine Leukocidin (PVL) was performed as previously published [16].

The SCC*mec *cassettes were first determined by a multiplex-PCR protocol previously described, and assigned to the corresponding types [30]. Furthermore, the results were confirmed by different multiplex-PCR protocols, focusing on the *mec *gene complex and the *ccr *gene complex [31].

### Molecular typing

All strains were grouped into clonal groups on the bases of their antibiotype, PFGE profile and SCC*mec *type, and representative isolates of the main PFGE sub-types A, B and E were further characterized by Multi Locus Sequence Typing (MLST), while all the other strains belonging to PFGE C, G, sporadic clones and 1980 strains were sequenced.

The clones were typed following the current nomenclature (ST-MRSA-SCC*mec*), except in those contexts in which maintaining the old nomenclature is necessary to understand the diversity existing in each MLST profile.

The sequences of the seven housekeeping genes used for MLST, corresponding to the allelic profile *arc*C-*aro*E-*glp*-*gmk*-*pta*-*tpi*-*yqi*L, were obtained by comparing the sequences obtained with those in the MLST database [32]. All STs described in the study were deposited on the MLST website and were compared with the major international *S. aureus *STs published.

### Statistical analysis

Glycopeptide MIC trends over the 17 years were assessed using non-parametric methods. Chi-square and Fisher's exact tests were performed and significance was defined as P < 0.001.

## Results

### Description of MRSA clones

The majority of MRSA clones in Italy, in agreement with the results of a previous study on strains isolated in 1990, belonged to six major clones: ST8-MRSA-I, ST247-MRSA-IA, ST239-MRSA-IIIA, ST228-MRSA-I, ST247-MRSA-I/IA and ST22-MRSA-IV and several minor clones. All these strains were previously typed by PFGE analysis (together with *Cla*I/*mec*A and *Cla*I/Tn*554 *and SCC*mec*), and grouped in six MRSA clones previously known as Archaic and Iberian (PFGE profile A, including, in both periods of time, 32 closely related sub-types), Brazilian (PFGE profile B, with 6 sub-types), Italian (PFGE profile E, with 10 sub-types), Rome (PFGE profile C) and Gentamicin-Susceptible (PFGE profile G).

**Figure 1:**
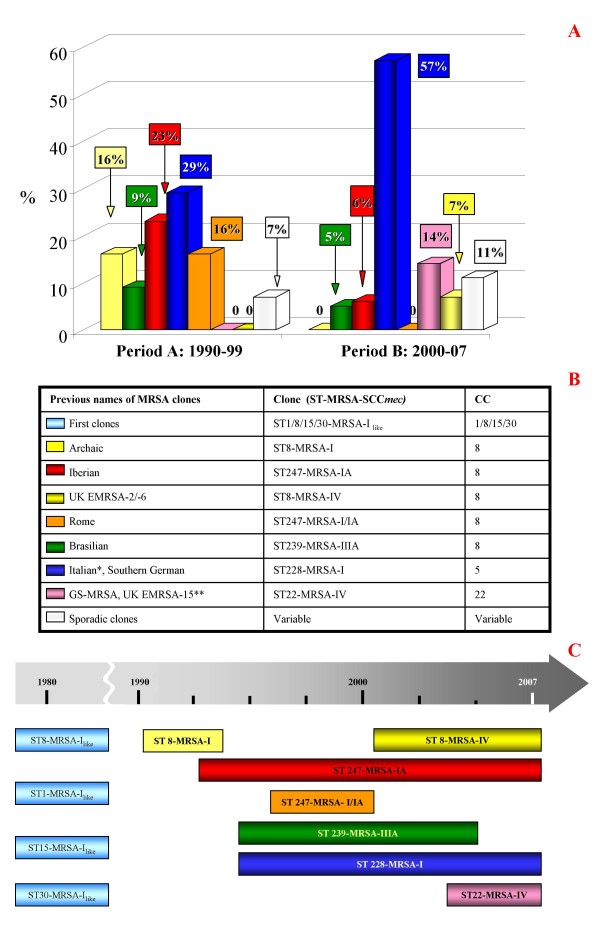
**Description of MRSA clones**. a) Distribution of the MRSA clones in periods A (1990–1999) and B (2000–2007); b) Description of the molecular backgrounds (ST, SCC*mec *and CC) of main clones isolated in Italy, correlated to their previous names; c) Graphical representation of alternation of MRSA clones in Italy during the 27-year period of study. *Italian and Southern Germany clones belong to the same ST228-MRSA-I, but they differ for their PFGE profile. **UK EMRSA-15 was often reported in literature as gentamicin-susceptible MRSA (GS-MRSA) clone. (ST: Sequence-Type; SCC*mec*: Staphylococcal Cassette Chromosome *mec*; CC: Clonal-Complex).

Figure 1a shows the distribution of the MRSA clones in the A and B periods covering seventeen years. The beginning of the 1990s was characterized by the presence (16% of isolates) of ST8, carrying the SCC*mec *type I. In 1993–1995, one major international epidemic clone, ST247-MRSA-IA, appeared in our hospitals (23%), together with ST228-MRSA-I circulating in our country (29%).

Another international clone, ST239-MRSA-IIIA, found to be resistant to cotrimoxazole, appeared in our hospitals around 1994 (9%), together with the local clone ST247-MRSA-I/IA, showing different phenotypic and genotypic features (erythromycin and clindamycin susceptibility; PFGE C), named Rome clone to differentiate it from the Iberian one. It appeared at the same time as ST239-MRSA-IIIA, but it has only been detected sporadically to date.

In 2000, we saw the decrease of ST247-IA (6% of isolates), the increase of ST228-I (57%), but also the re-appearance of ST8 (UK EMRSA-2/-6) carrying the SCC*mec*IV (7% of strains) instead of the SCC*mec*I, characteristic of the period between 1985–1990 (data not shown). Besides the major international clones, the isolation of a gentamicin-susceptible one, ST22-MRSA-IV, was detected in some hospitals, reaching 14% of isolates.

The *lukF*-PV and lukS-PV genes were detected in three CA-MRSA strains, belonging to ST8 and ST30, carrying SCC*mec *IV, and ST88, carrying SCC*mec *V.

The original and current nomenclature and molecular characteristics of each clone are shown in Figure 1b.

The group of strains isolated in 1980 was included for comparison (15 strains); ST8-MRSA-I (4 strains), remaining 11 strains belonged to ST1-MRSA-I (2 strains), ST15-MRSA-I (4 strains) and ST30-MRSA-I (5 strains), while of these 11 only 4 strains carried a variant of SCC*mec *type I. In particular those belonging to ST1-MRSA-I_like _(1 strain) and ST15-MRSA-I_like _(1 strain) carried *ccr*A_2_B_2 _recombinases instead of *ccr*A_1_B_1_, while ST30-MRSA-I_like _(2 strains) did not carry any recombinases genes (figure 1c). SCC*mec *multiplex PCR assays did not show any difference between the common type I (*pls*, *mec*A and *dcs *loci) and the variant. Recombinases multiplex PCR and LONG-PCR experiments in those strains carrying *ccr*A_2_B_2_recombinases, revealed a unusual fragment, of about 9 Kb, from the *pls *gene (*cif*F, primer up) to *ccr*A_2_B_2 _(*ccr*B2, primer dw).

Figure 1c also graphically represents the alternation of diverse clones over the 27 year time frame. Considering the strains isolated in 1980, it can be seen that, with the only exception of ST8, all the other clones disappeared from hospitals. In our results, this clone, i.e. the early ST8-MRSA-I (variable PFGE profiles), underwent a sort of evolution passing from an ST8-MRSA-I (PFGE A, with 3 sub-types) to the MDR ST247-MRSA-IA (PFGE A, with 28 sub-types) and back again to a more susceptible clone i.e the ST8-MRSA-IV (PFGE A, 1 sub-type) isolated in 2007. A characteristic of the clones at the end of period A and the beginning of period B is their multi-drug resistance, while the majority of period B saw a reduction in the number of these clones and a tendency towards susceptibility with the appearance of ST8-MRSA-IV and ST22-MRSA-IV – reflecting what was found in 1980, and indicating a possible future clinical change.

### Antibiotic susceptibilities

All the 301 MRSA strains were tested for their susceptibility to all classes of antibiotics. With the only exception of the strains isolated in 1980 that were uniformly susceptible to all non-beta lactam antibiotics, the remaining isolates were multi-resistant (Table 1). Both groups – A and B – acquired and maintained their level of resistance to gentamicin (98.1 and 88% respectively), erythromycin (83.1 versus 83.7%), clindamycin (85 and 75%) and ciprofloxacin (97 and 96.5). On the contrary, the isolates of group A were 67% resistant to tetracycline, while only 21% of group B isolates were resistant to this drug. The same decrease was observed for rifampin (from 66.2% to 21%) and cotrimoxazole (42 to 8.5%). The 15 MRSA strains from 1980 had from 73.3 to 86.6% susceptibility to all these drugs.

**Table 1:** Antibiotic-resistance (%) of various antibiotics against 301 MRSA strains, compared with a sample of Italian isolates from 1980.

**Antibiotics**	**MRSA isolated in 1980 (15)**		**MRSA isolated in 1990–1999 (period A) (160)**		**MRSA isolated in 2000–2007 (period B) (141)**	
	S	R	S	R	S	R
Gentamicin	73.3	26.6	1.9	98.1	22**	88
Erythromycin	86.6	13.3	16.9	83.1	16.3	83.7
Clindamycin	86.6	13.3	15	85	35	75
Tetracycline	73.3	26.6	33.1	67	82.2°	17.8
Ciprofloxacin	80	20	3	97	3.5	96.5
Rifampin	73.3	26.6	33.7	66.3*	79	21
Cotrimoxazole	86.6	13.3	58.1***	42	91.5	8.5

* Rifampin resistance is characteristic of CC8 (ST247 and 239)

** Including the gentamicin-susceptible clone

*** Cotrimoxazole resistance is a marker of ST 239

° The tetracycline susceptible Italian clone increased in this period

**Figure 2:**
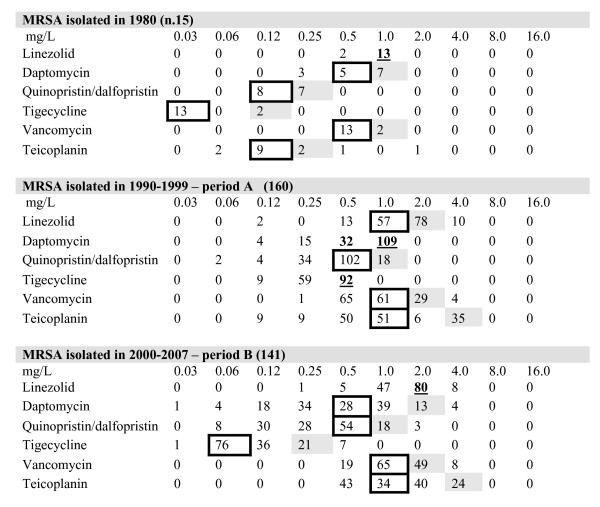
**MIC distributions of the major anti Gram-positive drugs against MRSA strains**. MIC_50 _and MIC_90 _in bold and underlined; MIC_50 _only in bold; MIC_90 _in grey.

The measure of the temporal shift in the susceptibility level of the anti-staphylococcal drugs, namely glycopeptides, linezolid, quinupristin/dalfopristin, daptomycin and tigecycline, was observed stratifying all the MIC data with respect to time. This distribution is shown in Figure 2. Making a comparison among MIC_90 _values, linezolid was stable in periods A and B with a MIC_90 _value of 2 mg/L; the same was true for quinupristin/dalfopristin (MIC_90 _of 1 mg/L in both periods), and for glycopeptides (2 and 4 mg/L for vancomycin and teicoplanin respectively). All isolates of the 1980 group were uniformly more susceptible to all antibiotics, as was predictable.

A MIC distribution evaluation demonstrated that the most striking change was a slight but continuous shift in vancomycin and teicoplanin MIC_s _over time. Table 2 shows this phenomenon in the three major clones, ST239-MRSA-IIIA, ST247-MRSA-IA and ST228-MRSA-I, isolated in Italy in periods A and B. While the percentage of strains with susceptibility to vancomycin of ≤ 0.5 mg/L was 52% in period A, this value decreased to 20.4% in period B with a corresponding increase in the number of strains with a MIC of 1 mg/L (44.1 versus 28.5%) and ≥ 2 mg/L (35.5% versus 19.4%). The same trend was observed for teicoplanin.

**Table 2:** Number of MRSA strains with glycopeptide MICs of ≤ 0.5, 1, and ≥ 2 mg/L in ST247-MRSA-IA, ST239-MRSA-IIIA and ST228-MRSA-I isolated in periods A and B

	**ST247-MRSA-IA, ST239-MRSA-IIIA and ST228-MRSA-I clones° 1990–1999 – period A (98)**			**ST247-MRSA-IA, ST239-MRSA-IIIA and ST228-MRSA-I clones° 2000–2007 – period B (93)**		
**MIC mg/L**	≤ 0.5	1	≥ 2	≤ 0.5	1	≥ 2
**Vancomycin (%)**	51 (52)	28 (28.5)	19 (19.4)	19 (20.4)	41 (44.1)	33 (35.5)
**Teicoplanin (%)**	49 (50)	25 (25.5)	22 (22.4)	20 (21.5)	30 (32.2)	43 (46.2)

° P < 0.001

Interestingly, this MIC creep was related to the parallel increase in the isolation of the MDR ST228-MRSA-I clone, in period B.

## Discussion

We studied 2 collections of MRSA strains identified over a 17-year period in Italy (periods A and B). These strains are representative of isolates collected during this time and published in two Italian surveys [24,25]. The results were compared with those obtained from a group of Italian MRSA strains isolated in 1980. This report describes the evolution of the major Italian clonal types and the evolution of their resistance to antibiotics. Due to the vast amount of data that we had collected every year, we used representative phenotypes of each year over the two survey periods.

The comparison of these data with those from 1980 gives important and unique insights into long-term MRSA evolution.

The presence of five major clones (ST8-MRSA-I, ST247-MRSA-IA, ST239-MRSA-IIIA, ST228-MRSA-I and ST247-MRSA-I/IA) was previously reported by Mato R. et al. in 1990 [7]. We demonstrate here that the prevalence of predominant clones changed over time and this change might have significant medical consequences, since the new clones often display different antibiotic resistance profiles. We also demonstrate that – during this time – there was a considerable increase in the prevalence of MDR clones, with the Italian one becoming predominant in our country. Furthermore, we demonstrate that the isolation of MRSA strains with a vancomycin MIC ≥ 2 mg/l is more frequent in the nosocomial-acquired strains of the B period, and that this shift is also present for teicoplanin.

### Epidemiological evolution of MRSA clones

All major circulating clones in Italy belonged to the 4 most common genetic background types (ST8, ST247, ST239, and ST228) and they have also been reported, for a long time, as main isolates from studies of HA-MRSA all around the world. Among these clones, ST228 was prevalently found in Europe and was first isolated in 1995 in South-Germany, Slovenia, Austria, and Italy [33,34]. This clone still represents, in our country, the most common HA-MRSA clone, it is not related to any other national clones, and its genetic background closely correlates with those of several *S.aureus *including MSSA, MRSA, and VISA, all belonging to the clonal complex 5 (CC5), confirming the role of horizontal transfer of *mec*A among different ancestral lineages, originating from a well adapted MSSA strain. In our country, this clone doubled its prevalence during the study period, accounting for more than half of the MRSA strains isolated in period B.

The evolutionary history of clones belonging to CC8 (ST8-MRSA-I, ST247-MRSA-IA, ST247-MRSA-I and IA, and ST8-MRSA-IV) is more complex. The acquisition of diverse SCC*mec *elements and/or other genetic elements, likely occurred in several occasions in this genetic background [8]. The few CC8 1980 clones in our study, shared SCC*mec *cassettes similar to SCC*mec *type I, probably their precursors.

During period A, we documented the evolution of the ST8-MRSA-I towards a more resistant phenotype, acquiring and integrating different genetic elements, in particular the plasmid pUB*110 *(carrying kanamycin and neomycin resistance genes) into the SCC*mec *region, giving the original type I cassette a more complex organization (SCC*mec *IA), and two copies of Tn*554 *(carrying erythromycin and spectinomycin resistance determinants) integrated in the genome. This clone, although being more resistant to antibiotics, maintained the same PFGE profile A (with diverse sub-types), and belongs to ST247, a single locus variant of ST8.

ST239-MRSA-IIIA had a short history in Italy, probably because it had come from other countries [35]. This clone is poorly represented in both periods A and B.

Period A was also characterized by the emergence of a local clone (Rome clone) possessing the same ST247 of the more diffused Iberian one, but with two different important characteristics: i) a different PFGE profile; ii) a predisposition to low level susceptibility to vancomycin, producing an hVISA phenotype [32]. The replacement of ST247 in recent years (period B) in our country has also involved this clone, which is now scarcely represented.

The decline, during period B, of ST247-MRSA-IA is documented in this study. Recent reports have shown that this clone was replaced by other pandemic clones [23,35-38]. The replacement of ST247-MRSA-IA might suggest that it had lost its epidemic potential during the last decade.

Also during period B, in which ST228-MRSA-I consolidated its presence and ST247 decreased, there was the re-appearance of ST8, carrying a different SCC*mec *type IV, that resembles the ancient one, being less resistant to antibiotics. In fact, the recent appearance of this clone is of interest, because it maintained a profile of antibiotic-susceptibility.

Finally, in the last two years of period B, different MRSA strains such as the gentamicin-susceptible ST22-MRSA-IV strain, appeared in different wards in some Italian hospitals. This appearance was accompanied, in the community, by the isolation of hyper-virulent, *pvl*-positive MRSA strains, belonging to ST8-MRSA-IV, ST30-MRSA-IV and ST88-MRSA-V, which we documented in Italy [15,39].

### Evolution of antibiotic-resistance

The multi-drug resistant phenotype is a particular characteristic of the methicillin-resistant *S.aureus *strains, almost related to the global presence and spread of MDR clones [14,40]. The homogeneous insusceptibility to all beta-lactams, characteristic of methicillin-resistant strains, together with the continuous accumulation and organization of many resistance genes, has made this species particularly difficult to treat. This diffused antibiotic-resistance is true for many classes of antibiotics such as aminoglycosides, macrolides, lincosamides, and fluoroquinolones, which, as demonstrated in this paper on contemporary isolates, appeared to maintain a low level of activity against MRSA. A notable exception seems to be the increased level of susceptibility to gentamicin found in MRSA isolated in period B, mainly due to the appearance, in our hospitals, of a gentamicin-susceptible clone, belonging to ST22.

The percentage of resistance to tetracycline, rifampin and cotrimoxazole, increased in period A, but decreased in period B: in this case the different antibiotic susceptibility patterns are linked to the epidemiologic change and replacement of clones with specific markers of resistance. In our study, the decrease in resistance to these three drugs is due to the replacement of the rifampin resistant ST247-MRSA-IA, ST239-MRSA-IIIA and ST247-MRSA-I/IA with the tetracycline susceptible ST228-MRSA-I [7].

The trend of susceptibility to the most used anti-MRSA drugs, as evaluated in our study, demonstrated that no resistance to vancomycin, teicoplanin, daptomycin, linezolid and quinupristin/dalfopristin was observed in the time frame of the study. Among these drugs, only daptomycin has recently been approved and marketed, explaining the substantial stability of the MIC_50 _and MIC_90 _over time. On the contrary, the evaluation of the MICs of the population of MRSA demonstrated something comparable to that observed for other classes of drugs: the strains isolated in period B, in which MDR clones were replaced by others with different resistance make-ups, show a distribution toward the lowest MIC concentrations. The same can be observed for quinupristin/dalfopristin.

Although reports of linezolid resistance in *S.aureus *are increasing [41,42] data on MIC creep are infrequent (Golan Y et al, Abstract from the 46^th ^ICAAC, San Francisco CA 2006; Abs C2-1157 p. 127). In our study, changes in distribution of susceptibility for linezolid are comparable in the two periods, demonstrating a one dilution increase in MIC only if compared with the susceptibility demonstrated during 1980. This creep towards higher levels of MICs is clear if glycopeptides are considered and mostly related to the increased isolation of the MDR ST228-MRSA-I clone in period B. This slight but continuous increase in the level of susceptibility of vancomycin was reported by many studies all around the world [14,43,44], many of them demonstrating a strict correlation between increased MIC values and therapeutic failure in infections sustained by MRSA [43,45,46]. The MIC creep phenomenon has produced conflicting results most likely due to the MIC statistics used [47,48]. Reports from large multicenter studies have not demonstrated changes in vancomycin susceptibilities over time [44]. However, these types of studies are not designed to detect subtle changes in MICs. As reported by other investigators, we demonstrate a statistically significant increase in MRSA vancomycin and teicoplanin MICs over time. The magnitude of this increase was similar in both drugs, but the largest increase was observed for vancomycin, in which strains showing a MIC > 1 mg/L increased from 47 to 78%.

In conclusion, we document here the alternation of multi-drug resistant MRSA clones in the 1990–2007 period, with the establishment of ST228-MRSA-I in our country, with respect to the clones present in 1980. The increase of the glycopeptide MIC levels, reflecting a worldwide trend, was also documented, due to the massive use of these drugs in clinical practice. We detected ST1, ST8, ST15, and ST30 in the 1980 isolates; thus we hypothesize their re- appearance as backgrounds for the current CA-MRSA clones, due to mechanisms as yet unknown.

## Competing interests

The authors declare that they have no competing interests.

## Authors' contributions

FC participated in the study design, molecular typing, genetic relationships among strains, interpretation of the results, and co-drafted the manuscript; DB participated in the study design, co-performing DNA extraction and PCR experiments, including SCC*mec *multiplex PCR and MLST; SB participated in the study design, co-performing phenotypic characterization and *in vitro *susceptibility testing of antibiotics; SS participated in the study design, interpretation of the data, co-drafted the manuscript and participated in the final revision.

## References

[B1] Stefani S, Varaldo PE (2003). Epidemiology of methicillin-resistant staphylococci in Europe. Clin Microbiol Infect.

[B2] Luzzaro F, Viganò EF, Fossati D, Grossi A, Sala A, Sturla C, Saudelli M, Toniolo A, AMCLI Lombardia Hospital Infectious Study Group (2002). Prevalence and drug susceptibility of pathogens causing bloodstream infections in northern Italy: a two-year study in 16 hospitals. Eur J Clin Microbiol Infect Dis.

[B3] Pan A, Carnevale G, Catenazzi P, Colombini P, Crema L, Dolcetti L, Ferrari L, Mondello P, Signorini L, Tinelli C, Roldan EQ, Carosi G (2005). Trends in methicillin-resistant Staphylococcus aureus (MRSA) bloodstream infections: effect of the MRSA "search and isolate" strategy in a hospital in Italy with hyperendemic MRSA. Infect Control Hosp Epidemiol.

[B4] Raineri E, Crema L, De Silvestri A, Acquarolo A, Albertario F, Carnevale G, Latronico N, Petrosillo N, Tinelli C, Zoncada A, Pan A (2007). Methicillin-resistant Staphylococcus aureus control in an intensive care unit: a 10 year analysis. J Hosp Infect.

[B5] Rodloff AC, Leclercq R, Debbia EA, Cantón R, Oppenheim BA, Dowzicky MJ (2008). Comparative analysis of antimicrobial susceptibility among organisms from France, Germany, Italy, Spain and the UK as part of the tigecycline evaluation and surveillance trial. Clin Microbiol Infect.

[B6] Robinson DA, Enright MC (2004). Multilocus sequence typing and the evolution of methicillin-resistant Staphylococcus aureus. Clin Microbiol Infect.

[B7] Mato R, Campanile F, Stefani S, Crisóstomo MI, Santagati M, Sanches SI, de Lencastre H (2004). Clonal types and multidrug resistance patterns of methicillin-resistant Staphylococcus aureus (MRSA) recovered in Italy during the 1990s. Microb Drug Resist.

[B8] Enright MC, Robinson DA, Randle G, Feil EJ, Grundmann H, Spratt BG (2002). The evolutionary history of methicillin-resistant Staphylococcus aureus (MRSA). Proc Natl Acad Sci USA.

[B9] Feil EJ, Li BC, Aanensen DM, Hanage WP, Spratt BG (2004). eBURST: inferring patterns of evolutionary descent among clusters of related bacterial genotypes from multilocus sequence typing data. J Bacteriol.

[B10] Holden MT, Feil EJ, Lindsay JA, Peacock SJ, Day NP, Enright MC, Foster TJ, Moore CE, Hurst L, Atkin R, Barron A, Bason N, Bentley SD, Chillingworth C, Chillingworth T, Churcher C, Clark L, Corton C, Cronin A, Doggett J, Dowd L, Feltwell T, Hance Z, Harris B, Hauser H, Holroyd S, Jagels K, James KD, Lennard N, Line A, Mayes R, Moule S, Mungall K, Ormond D, Quail MA, Rabbinowitsch E, Rutherford K, Sanders M, Sharp S, Simmonds M, Stevens K, Whitehead S, Barrell BG, Spratt BG, Parkhill J (2004). Complete genomes of two clinical Staphylococcus aureus strains: evidence for the rapid evolution of virulence and drug resistance. Proc Natl Acad Sci USA.

[B11] Diep BA, Gill SR, Chang RF, Phan TH, Chen JH, Davidson MG, Lin F, Lin J, Carleton HA, Mongodin EF, Sensabaugh GF, Perdreau-Remington F (2006). Complete genome sequence of USA300, an epidemic clone of community-acquired meticillin-resistant Staphylococcus aureus. Lancet.

[B12] Lindsay JA, Moore CE, Day NP, Peacock SJ, Witney AA, Stabler RA, Husain SE, Butcher PD, Hinds J (2006). Microarrays reveal that each of the ten dominant lineages of Staphylococcus aureus has a unique combination of surface-associated and regulatory genes. J Bacteriol.

[B13] Hope R, Livermore DM, Brick G, Lillie M, Reynolds R, BSAC Working Parties on Resistance Surveillance (2008). Non-susceptibility trends among staphylococci from bacteraemias in the UK and Ireland, 2001–06. J Antimicrob Chemother.

[B14] Sakoulas G, Moellering RC (2008). Increasing antibiotic resistance among methicillin-resistant Staphylococcus aureus strains. Clin Infect Dis.

[B15] Valentini P, Parisi G, Monaco M, Crea F, Spanu T, Ranno O, Tronci M, Pantosti A (2008). An uncommon presentation for a severe invasive infection due to methicillin-resistant Staphylococcus aureus clone USA300 in Italy: a case report. Ann Clin Microbiol Antimicrob.

[B16] Stefani S, Bongiorno D, Cafiso V, Campanile F, Crapis M, Cristini F, Sartor A, Scarparo C, Spina D, Viale P (2009). Pathotype and susceptibility profile of a community-acquired methicillin-resistant Staphylococcus aureus strain responsible for a case of severe pneumonia. Diagn Microbiol Infect Dis.

[B17] Enright MC (2003). The evolution of a resistant pathogen–the case of MRSA. Current opinion in pharmacology.

[B18] Rossney AS, Lawrence MJ, Morgan PM, Fitzgibbon MM, Shore A, Coleman DC, Keane CT, O'Connell B (2006). Epidemiological typing of MRSA isolates from blood cultures taken in Irish hospitals participating in the European Antimicrobial Resistance Surveillance System (1999–2003). Eur J Clin Microbiol Infect Dis.

[B19] Conceição T, Aires-de-Sousa M, Füzi M, Tóth A, Pászti J, Ungvári E, van Leeuwen WB, van Belkum A, Grundmann H, de Lencastre H (2007). Replacement of methicillin-resistant Staphylococcus aureus clones in Hungary over time: a 10-year surveillance study. Clin Microbiol Infect.

[B20] Francois P, Harbarth S, Huyghe A, Renzi G, Bento M, Gervaix A, Pittet D, Schrenzel J (2008). Methicillin-resistant Staphylococcus aureus, Geneva, Switzerland, 1993–2005. Emerg Infect Dis.

[B21] Kerttula AM, Lyytikäinen O, Kardén-Lilja M, Ibrahem S, Salmenlinna S, Virolainen A, Vuopio-Varkila J (2007). Nationwide trends in molecular epidemiology of methicillin-resistant Staphylococcus aureus, Finland, 1997–2004. BMC Infect Dis.

[B22] Vindel A, Trincado P, Gómez E, Cabrera R, Boquete T, Solá C, Valdezate S, Saez-Nieto JA (2006). Prevalence and evolution of methicillin-resistant Staphylococcus aureus in Spanish hospitals between 1996 and 2002. J Clin Microbiol.

[B23] Blanc DS, Petignat C, Wenger A, Kuhn G, Vallet Y, Fracheboud D, Trachsel S, Reymond M, Troillet N, Siegrist HH, Oeuvray S, Bes M, Etienne J, Bille J, Francioli P, Zanetti G (2007). Changing molecular epidemiology of methicillin-resistant Staphylococcus aureus in a small geographic area over an eight-year period. J Clin Microbiol.

[B24] Stefani S, Mezzatesta ML, Tempera G, Debbra E, Schito AM, Nicoletti G, Marchére A (2002). Comparative activity of linezolid against staphylococci and enterococci isolated in Italy. Clin Microbiol Infect.

[B25] Nicoletti G, Schito G, Fadda G, Boros S, Nicolosi D, Marchese A, Spanu T, Pantosti A, Monaco M, Rezza G, Cassone A, Garaci E, CIGAR (Gruppo Cooperativo Infezioni Gravi ed Antibiotico Resistenza) (2006). Bacterial isolates from severe infections and their antibiotic susceptibility patterns in Italy: a nationwide study in the hospital setting. J Chemother.

[B26] Varaldo PE, Cipriani P, Focá A, Geraci C, Giordano A, Madeddu MA, Orsi A, Pompei R, Prenna M, Repetto A (1984). Identification, clinical distribution, and susceptibility to methicillin and 18 additional antibiotics of clinical Staphylococcus isolates: nationwide investigation in Italy. J Clin Microbiol.

[B27] Clinical Laboratory Standards, C.L.S.I (2008). Performance Standards for Antimicrobial Susceptibility Testing – Approved Standard.

[B28] Campanile F, Bongiorno D, Borbone S, Venditti M, Giannella M, Franchi C, Stefani S (2007). Characterization of a variant of the SCCmec element in a bloodstream isolate of Staphylococcus intermedius. Microb Drug Resist.

[B29] Tenover FC, Arbeit RD, Goering RV, Mickelsen PA, Murray BE, Persing DH, Swaminathan B (1995). Interpreting chromosomal DNA restriction patterns produced by pulsed- field gel electrophoresis: criteria for bacterial strain typing. J Clin Microbiol.

[B30] Oliveira DC, de Lencastre H (2002). Multiplex PCR strategy for rapid identification of structural types and variants of the mec element in methicillin-resistant Staphylococcus aureus. Antimicrob Agents Chemother.

[B31] Kondo Y, Ito T, Ma XX, Watanabe S, Kreiswirth BN, Etienne J, Hiramatsu K (2007). Combination of multiplex PCRs for staphylococcal cassette chromosome mec type assignment: rapid identification system for mec, ccr, and major differences in junkyard regions. Antimicrob Agents Chemother.

[B32] Cassone M, Campanile F, Pantosti A, Venditti M, Stefani S (2004). Identification of a variant "Rome clone" of methicillin-resistant Staphylococcus aureus with decreased susceptibility to vancomycin, responsible for an outbreak in an intensive care unit. Microb Drug Resist.

[B33] Krziwanek K, Luger C, Sammer B, Stumvoll S, Stammler M, Sagel U, Witte W, Mittermayer H (2008). MRSA in Austria–an overview. Clin Microbiol Infect.

[B34] Deurenberg RH, Vink C, Kalenic S, Friedrich AW, Bruggeman CA, Stobberingh EE (2007). The molecular evolution of methicillin-resistant Staphylococcus aureus. Clin Microbiol Infect.

[B35] Amorim ML, Aires de Sousa M, Sanches IS, Sá-Leão R, Cabeda JM, Amorim JM, de Lencastre H (2002). Clonal and antibiotic resistance profiles of methicillin-resistant Staphylococcus aureus (MRSA) from a Portuguese hospital over time. Microb Drug Resist.

[B36] Teixeira LA, Resende CA, Ormonde LR, Rosenbaum R, Figueiredo AM, de Lencastre H, Tomasz A (1995). Geographic spread of epidemic multiresistant Staphylococcus aureus clone in Brazil. J Clin Microbiol.

[B37] Cookson BD, Phillips I (1988). Epidemic methicillin-resistant Staphylococcus aureus. J Antimicrob Chemother.

[B38] Pérez-Roth E, Lorenzo-Díaz F, Batista N, Moreno A, Méndez-Alvarez S (2004). Tracking methicillin-resistant Staphylococcus aureus clones during a 5-year period (1998 to 2002) in a Spanish hospital. J Clin Microbiol.

[B39] Monaco M, Antonucci R, Palange P, Venditti M, Pantosti A (2005). Methicillin-resistant Staphylococcus aureus necrotizing pneumonia. Emerg Infect Dis.

[B40] Deurenberg RH, Stobberingh EE (2008). The evolution of Staphylococcus aureus. Infect Genet Evol.

[B41] Gales AC, Sader HS, Andrade SS, Lutz L, Machado A, Barth AL (2006). Emergence of linezolid-resistant Staphylococcus aureus during treatment of pulmonary infection in a patient with cystic fibrosis. Int J Antimicrob Agents.

[B42] Tsiodras S, Gold HS, Sakoulas G, Eliopoulos GM, Wennersten C, Venkataraman L, Moellering RC, Ferraro MJ (2001). Linezolid resistance in a clinical isolate of Staphylococcus aureus. Lancet.

[B43] Soriano A, Marco F, Martínez JA, Pisos E, Almela M, Dimova VP, Alamo D, Ortega M, Lopez J, Mensa J (2008). Influence of vancomycin minimum inhibitory concentration on the treatment of methicillin-resistant Staphylococcus aureus bacteremia. Clin Infect Dis.

[B44] Steinkraus G, White R, Friedrich L (2007). Vancomycin MIC creep in non-vancomycin-intermediate Staphylococcus aureus (VISA), vancomycin-susceptible clinical methicillin-resistant S. aureus (MRSA) blood isolates from 2001–05. J Antimicrob Chemother.

[B45] Sakoulas G, Gold HS, Cohen RA, Venkataraman L, Moellering RC, Eliopoulos GM (2006). Effects of prolonged vancomycin administration on methicillin-resistant Staphylococcus aureus (MRSA) in a patient with recurrent bacteraemia. J Antimicrob Chemother.

[B46] Sakoulas G, Moise-Broder PA, Schentag J, Forrest A, Moellering RC, Eliopoulos GM (2004). Relationship of MIC and bactericidal activity to efficacy of vancomycin for treatment of methicillin-resistant Staphylococcus aureus bacteremia. J Clin Microbiol.

[B47] Castanheira M, Jones R, DSader H (2008). Update of the in vitro activity of daptomycin tested against 6710 Gram-positive cocci isolated in North America (2006). Diagn Microbiol Infect Dis.

[B48] Alós JI, García-Cañas A, García-Hierro P, Rodríguez-Salvanés F (2008). Vancomycin MICs did not creep in Staphylococcus aureus isolates from 2002 to 2006 in a setting with low vancomycin usage. J Antimicrob Chemother.

